# Aminophylline and Ephedrine, but Not Flumazenil, Inhibit the Activity of the Excitatory Amino Acid Transporter 3 Expressed in* Xenopus* Oocytes and Reverse the Increased Activity by Propofol

**DOI:** 10.1155/2018/6817932

**Published:** 2018-05-20

**Authors:** Sohyeon Moon, Hee Jung Baik

**Affiliations:** ^1^Graduate School of Pharmaceutical Sciences, Ewha Womans University, 52 Ewhayeodae-gil, Seodaemun-gu, Seoul 03760, Republic of Korea; ^2^Department of Anesthesiology and Pain Medicine, College of Medicine, Ewha Womans University, 1071 An Yang Cheon Ro, Yang Cheon Gu, Seoul 07985, Republic of Korea

## Abstract

We investigated the effects of flumazenil, aminophylline, and ephedrine on the excitatory amino acid transporter type 3 (EAAT3) activity and the interaction with propofol. EAAT3 was expressed in the* Xenopus* oocytes. L-Glutamate-induced membrane currents were measured using the two-electrode voltage clamp at various drug concentrations. Oocytes were preincubated with protein kinase C- (PKC-) activator, or inhibitor, and phosphatidylinositol 3-kinase (PI3K) inhibitor. To study the interaction with propofol, oocytes were exposed to propofol, propofol + aminophylline, or ephedrine. Aminophylline and ephedrine significantly decreased EAAT3 activity. Aminophylline (95 *μ*M) and ephedrine (1.19 *μ*M) significantly decreased *V*max, but not Km of EAAT3, for glutamate. The phorbol 12-myristate-13-acetate-induced increase in EAAT3 activity was abolished by aminophylline or ephedrine. The decreased EAAT3 activities by PKC inhibitors (staurosporine, chelerythrine) and PI3K inhibitor (wortmannin) were not significantly different from those by aminophylline or ephedrine, as well as those by PKC inhibitors or PI3K inhibitor + aminophylline or ephedrine. The enhanced EAAT3 activities induced by propofol were significantly abolished by aminophylline or ephedrine. Aminophylline and ephedrine inhibit EAAT3 activity via PKC and PI3K pathways and abolish the increased EAAT3 activity by propofol. Our results indicate a novel site of action for aminophylline and ephedrine.

## 1. Introduction

Glutamate is a major excitatory neurotransmitter, and glutamate transporters play a critical role in maintaining extracellular glutamate concentrations by the uptake of extracellular glutamate into the intracellular spaces, thereby regulating normal excitatory neurotransmission and preventing excitotoxicity [1–3]. Of five glutamate transporters, excitatory amino acid transporter type 3 (EAAT3) is the major neuronal transporter in the brain and spinal cord and plays a neuroprotective role by maintaining extracellular glutamate homeostasis in the cortex and hippocampus, which are vulnerable to excitotoxic damage after brain ischemia [1, 4].

The effects of anesthetics on EAAT3 activity have been investigated. Do et al. reported that volatile anesthetics, as well as propofol at clinically relevant concentrations, increased the EAAT3 activity via protein kinase C (PKC) activation [5, 6]. Their results suggest that the enhanced EAAT3 activity by inhalational anesthetics or propofol may be an important mechanism for their anesthetic, neuroprotective, and antiepileptic effects [5, 6].

Considering the enhanced EAAT3 activity as a new target for anesthetic effect, some drugs antagonizing sedative and anesthetic effects may affect EAAT3 activity and reverse the increased activity induced by anesthetics. It has been reported that flumazenil, a specific benzodiazepine receptor antagonist, has intrinsic actions. Flumazenil reversed hepatic coma in humans [7] and increased the degree of anxiety [8]. Aminophylline, a nonselective adenosine receptor antagonist, blocks the action of adenosine in central adenosinergic neuromodulator systems [9], has excitatory effects on neuronal activity, and stimulates the central nervous system (CNS) to induce vigilance [10]. The roles of adenosine in sleep and its sedative/hypnotic activities are well known [11]. Many reports have suggested that aminophylline antagonizes the sedative effects of inhalational and intravenous anesthetics [12–15]. Ephedrine, a vasopressor, also has potent stimulating effects on CNS. There are several studies suggesting that ephedrine can change depth of anesthesia in animals and human. Ephedrine increases minimum alveolar concentration (MAC) of halothane in dogs [16] and increases bispectral index (BIS) during sevoflurane or propofol anesthesia in human [17, 18], both of which suggest its antagonistic action to anesthetic effects. Adenosine and dopamine are important modulators of glutamatergic neurotransmission in the striatum [19]. These findings suggest that flumazenil, aminophylline, and ephedrine may affect glutamate transporter activity and show interactions with propofol on their effects on EAAT3 activities. However, the mechanism on the excitatory action of these drugs is unclear and little is known about the effects of these drugs on EAAT3 activities and their interactions with increased EAAT3 activity induced by propofol.

In the present study, we hypothesized that flumazenil, aminophylline, and ephedrine may affect EAAT3 activity. To elucidate the mechanism on the excitatory action of flumazenil, aminophylline, and ephedrine, we investigated the effects of these three drugs on EAAT3 activity, and the involvement of protein kinase C (PKC) and phosphatidylinositol 3-kinase (PI3K), two important modulating kinases in intracellular signaling, in their effects on EAAT3. We also investigated the interaction with propofol with regard to their effects on EAAT3 activity.

## 2. Materials and Methods

The protocol for this animal experiment was approved by the Institutional Animal Care and Use Committee at the School of Medicine, Ewha Womans University (Seoul, Republic of Korea; Approval Number ESM 11-0181, September 1, 2011). The experiments were conducted at the research laboratories of the School of Medicine, Ewha Womans University. The study was performed according to the Declaration of Helsinki and internationally accepted principles in the care and use of experimental animals. Mature female frogs* (Xenopus laevis)* were purchased from Xenopus I (Dexter, MI, USA). Unless otherwise noted, all reagents were acquired from Sigma (St. Louis, MO, USA).

### 2.1. Oocyte Preparation, Microinjection, and Electrophysiological Recordings

Oocyte preparation and microinjection of EAAT3 mRNA into oocytes were performed as described previously [5, 20]. Rat EAAT3 complementary DNA construct was supplied by Dr. Mattias A. Hediger (Brigham and Women's Hospital, Harvard Institutes of Medicine, Boston, MA, USA). The prepared oocytes were then incubated at 16°C in modified Barth's solution for 3 days before the voltage clamp experiments. The composition of modified Barth's solution was the same as described in previous reports [5, 20].

Voltage clamp experiments were also performed as described previously [20] at room temperature (approximately 21°C–23°C). Analyses were carried out using pCLAMP7 software (Axon Instruments, Foster City, CA, USA). All measurements were performed at a holding potential of −70 mV. Oocytes showing an unstable holding current ≥1 *μ*A were excluded from the analysis. l-Glutamate diluted in Tyrode's solution was perfused over the oocytes for 25 s (5 mL/min). l-Glutamate-induced inward currents were recorded at 125 Hz for 1 min: 5 s of baseline, 25 s of l-glutamate application, and a 30-s rinse with Tyrode's solution, the composition of which was the same as that described previously [20]. The glutamate-induced peak currents were calculated to reflect the amount of glutamate transported. Unless otherwise indicated, we used 30 *μ*M l-glutamate in the study based on the value of the pharmacokinetic parameter *K*_*m*_ of EAAT3 for l-glutamate of 27–30 *μ*M [5, 21].

### 2.2. Administration of Experimental Chemicals

Flumazenil dissolved in methanol was diluted with Tyrode's solution to the appropriate concentration (16 nM, 66 nM, 264 nM, 528 nM, and 1.32 *μ*M), which included clinically relevant plasma concentrations [22]. Aminophylline (2.5%) (Dai Han Pharm. Co., Ltd., Seoul, Korea) and ephedrine (Jeil Pharmaceutical Co., Ltd., Daegu, Korea) were diluted with Tyrode's solution to the appropriate concentration (aminophylline: 9.5, 23.8, 47.6, 95.1, and 237.9 *μ*M; ephedrine: 50 nM, 99 nM, 298 nM, 595 nM, 1.19 *μ*M, 2.48 *μ*M, and 4.96 *μ*M), which included clinically pertinent plasma concentrations of each drug [23–26]. In control experiments, we perfused oocytes with Tyrode's solution for 4 min before applying l-glutamate in Tyrode's solution for electrophysiological recording. In the drug group, we perfused oocytes with Tyrode's solution for 1 minute to allow stabilization followed by Tyrode's solution containing the test drug for 3 min before application of Tyrode's solution containing l-glutamate. To determine the effects of aminophylline and ephedrine on the *K*_*m*_ and *V*_max⁡ _ values of EAAT3 for glutamate, serial concentrations of l-glutamate (3, 10, 30, 100, and 300 *μ*M) were used.

To study the effects of PKC activation on EAAT3 activity, oocytes were preincubated with 100 nM phorbol 12-myristate-13-acetate (PMA), a PKC activator, for 10 min before recording [27]. As flumazenil did not show any significant effects on EAAT3 activity at any tested concentrations, we excluded flumazenil in this study. To investigate whether there is interaction between PMA and aminophylline or ephedrine, PMA-treated oocytes were exposed to 95.1 *μ*M aminophylline or 1.19 *μ*M ephedrine as described above. These concentrations are within therapeutic ranges of aminophylline and ephedrine. To study the role of PKC inhibitors on EAAT3 activity, oocytes were preincubated with the PKC inhibitors, 2 *μ*M staurosporine (for 1 h) and 100 *μ*M chelerythrine (for 1 h) just before the application of 95.1 *μ*M aminophylline or 1.19 *μ*M ephedrine as described above [28, 29]. To investigate the role of PI3K in the regulation of EAAT3 activity, oocytes were incubated with wortmannin (10 *μ*M for 1 h) and then exposed to 95.1 *μ*M aminophylline or 1.19 *μ*M ephedrine as described above [29].

**Figure 1:**
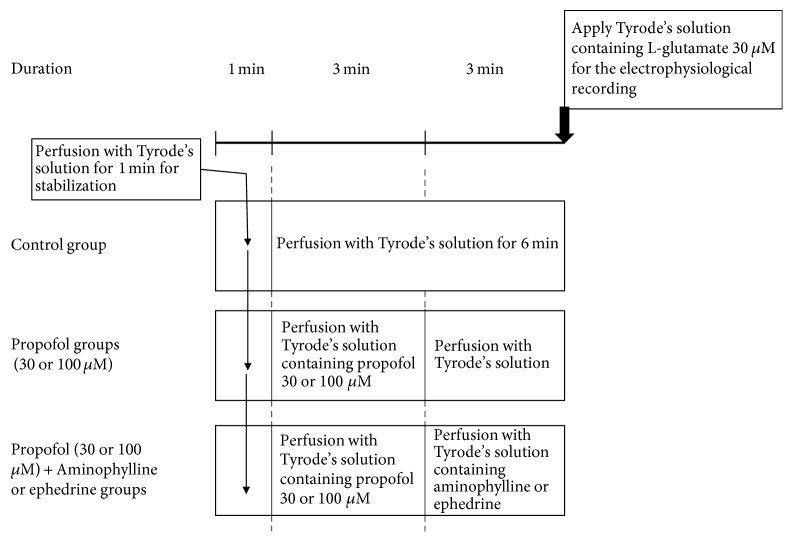
Schematic representation of the protocol used in the experiment for interactions between the effects of aminophylline or ephedrine and propofol on EAAT3 activity.

To study the interaction with propofol, we used propofol (Fresofol MCT 1%) (Fresenius Kabi Austria, Graz, Austria) at concentrations of 30 and 100 *μ*M [6]. In a preliminary experiment to establish the protocol for interaction experiments between propofol and drugs (aminophylline or ephedrine), we found that both oocytes perfused with Tyrode's solution for 1 min followed by Tyrode's solution containing 30 *μ*M propofol for 3 min (propofol group, 1.29 ± 0.07) and oocytes perfused with Tyrode's solution for 1 min followed by Tyrode's solution containing 30 *μ*M propofol for 3 min and then Tyrode's solution for 3 min (propofol + Tyrode's solution group, 1.26 ± 0.05) significantly increased EAAT3 activity, compared with the control group (1.00  ±  0.05) (*n* = 17-18, *P* < 0.05). However, there was no significant difference in EAAT3 activity between the propofol group and the propofol + Tyrode's solution group (*P* > 0.05). Therefore, we determined the protocol for the interaction experiment, as shown in Figure 1. In the control group, we perfused oocytes with Tyrode's solution for 7 min before applying Tyrode's solution containing l-glutamate for recording. In the 30 and 100 *μ*M propofol groups, we perfused oocytes with Tyrode's solution for 1 min followed by Tyrode's solution containing propofol for 3 min, and then Tyrode's solution for 3 min before applying l-glutamate. In the propofol (30 or 100 *μ*M) + aminophylline (95.1 *μ*M) or ephedrine (1.19 *μ*M) groups, we perfused oocytes with Tyrode's solution for 1 min followed by Tyrode's solution containing propofol for 3 min, and then Tyrode's solution containing aminophylline or ephedrine for 3 min before applying l-glutamate.

### 2.3. Statistical Analysis

Responses are shown as means ± SEM. Each experiment was performed with oocytes from at least three different frogs. As the EAAT3 expression levels in oocytes of different batches may vary, variability in the responses of oocytes among batches is common. Therefore, responses were normalized to the mean value of the same-day controls for each batch. Statistical analysis was done using a one-way analysis of variance followed by Dunnett's or Tukey's multiple comparison test and the Mann–Whitney test as appropriate (SPSS version 20.0; SPSS Inc., Chicago, IL, USA). *P* < 0.05 was considered significant. Dose-response curves were drawn using GraphPad Prism 5.0 (GraphPad Software, San Diego, CA, USA). Concentration-response curves of EAAT3 to l-glutamate in the presence or absence of aminophylline and ephedrine were made, and *V*_max_ and *K*_*m*_ were calculated using SigmaPlot 12.0 (Systat Software Inc., San Jose, CA, USA).

## 3. Results

**Figure 2:**
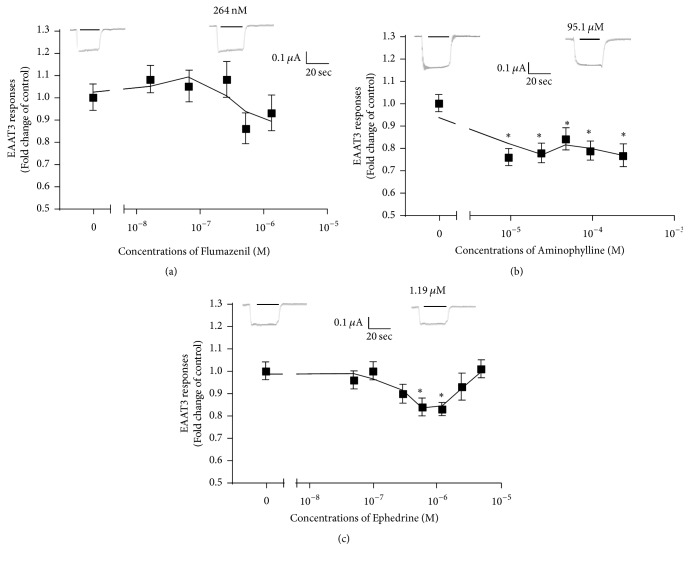
Dose-response curves of the effects of flumazenil (a), aminophylline (b), and ephedrine (c) on EAAT3 responses to l-glutamate (30 *μ*M). Data are means ± SEM,* n* = 26–38 (a), 27–36 (b), and 41–60 (c) for each data point.  ^*∗*^*P* < 0.05 compared with the control.

Oocytes that were not injected were not responsive to l-glutamate (data not shown); those injected with EAAT3 mRNA induced inward currents by application of 30 *μ*M l-glutamate (Figure 2). This current was demonstrated to be mediated by EAAT3 in previous studies [5, 27]. The highest concentration of methanol, the solvent for flumazenil, in Tyrode's solution containing flumazenil was 0.04% (v/v). Our previous study showed that the current induced by glutamate in oocytes expressing EAAT3 was unaffected by this concentration of methanol [27].

While flumazenil, aminophylline, or ephedrine alone did not generate any current in oocytes with or without EAAT3 mRNA (data not shown), aminophylline significantly decreased EAAT3 responses to l-glutamate at all concentrations studied (9.5–238 *μ*M), but ephedrine did so only at 595 nM and 1.19 *μ*M (*P* < 0.05) (Figure 2). However, flumazenil did not significantly affect EAAT3 responses at any concentration tested (0.016–1.319 *μ*M) (*P* > 0.05) (Figure 2). Based on the dose-response results for these drugs, we used 95.1 *μ*M aminophylline and 1.19 *μ*M ephedrine in subsequent experiments.

**Figure 3:**
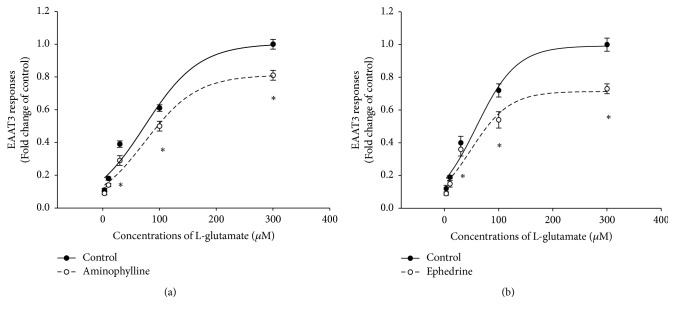
Concentration-response curves of excitatory amino acid transporter type 3 (EAAT3) to l-glutamate in the presence or absence of 95.1 *μ*M aminophylline (a) and 1.19 *μ*M ephedrine (b). Data are means ± SEM,* n* = 8–28 for each data point. ^*∗*^*P* < 0.05 compared with the corresponding controls.

Together with decreasing the responses caused by 30 *μ*M l-glutamate, 95.1 *μ*M aminophylline-treated and 1.19 *μ*M ephedrine-treated oocytes also showed significantly decreased responses to 100 or 300 *μ*M l-glutamate (Figures 3(a) and 3(b)). Further analyses showed that aminophylline and ephedrine significantly reduced *V*_max_ (1.00 ± 0.10 for control versus 0.81 ± 0.07 for aminophylline, and 0.99 ± 0.08 for control versus 0.71 ± 0.09 for ephedrine, *P* < 0.05), but not *K*_*m*_ of EAAT3 for l-glutamate (74.1 ± 18.5 *μ*M for control versus 74.8 ± 14.4 *μ*M for aminophylline, and 58.5 ± 12.2 *μ*M for control versus 48.8 ± 18.3 *μ*M for ephedrine, *P* > 0.05).

**Figure 4:**
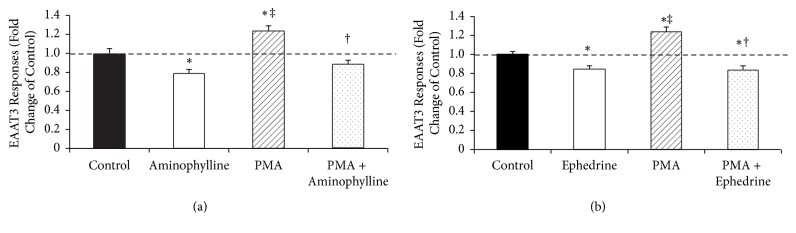
Effects of protein kinase C (PKC) activation on excitatory amino acid transporter type 3 (EAAT3) activity in the presence or absence of 95.1 *μ*M aminophylline (a) and 1.19 *μ*M ephedrine (b). PMA, phorbol 12-myristate 13-acetate. Data are means ± SEM,* n* = 22–46 for each data point. ^*∗*^*P* < 0.05 compared with control; ^†^*P* < 0.05 compared with PMA alone; ^‡^*P* < 0.05 compared with aminophylline or ephedrine alone.

PMA (100 nM for 10 min) increased the EAAT3 activity significantly (1.00 ± 0.05 for control versus 1.24 ± 0.05 for PMA, *P* < 0.05). When PMA-treated (100 nM for 10 min) oocytes were exposed to aminophylline (95.1 *μ*M) or ephedrine (1.19 *μ*M), the PMA-induced increase in EAAT3 activity was abolished (0.89 ± 0.04 for PMA + aminophylline; 0.84 ± 0.04 for PMA + ephedrine, *P* < 0.05 compared with PMA) (Figures 4(a) and 4(b)).

**Figure 5:**
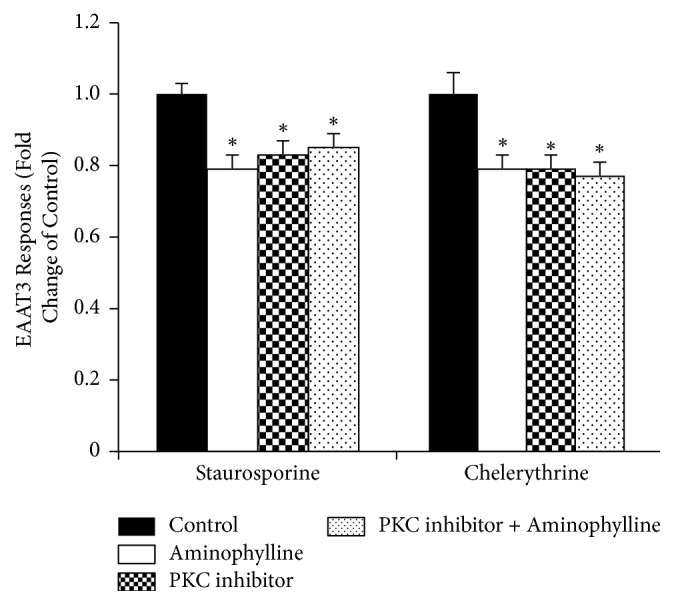
Effects of protein kinase C (PKC) inhibition on excitatory amino acid transporter type 3 (EAAT3) activity in the presence or absence of 95.1 *μ*M aminophylline. Whereas oocytes exposed to PKC inhibitor, aminophylline, or both showed a significant decrease in EAAT3 activity by 30 *μ*M glutamate compared with the control, the EAAT3 activities were not significantly different among oocytes treated with PKC inhibitor, aminophylline, or PKC inhibitor plus aminophylline. Data are means ± SEM,* n* = 31–41 for each data point. ^*∗*^*P* < 0.05 compared with the control.

**Figure 6:**
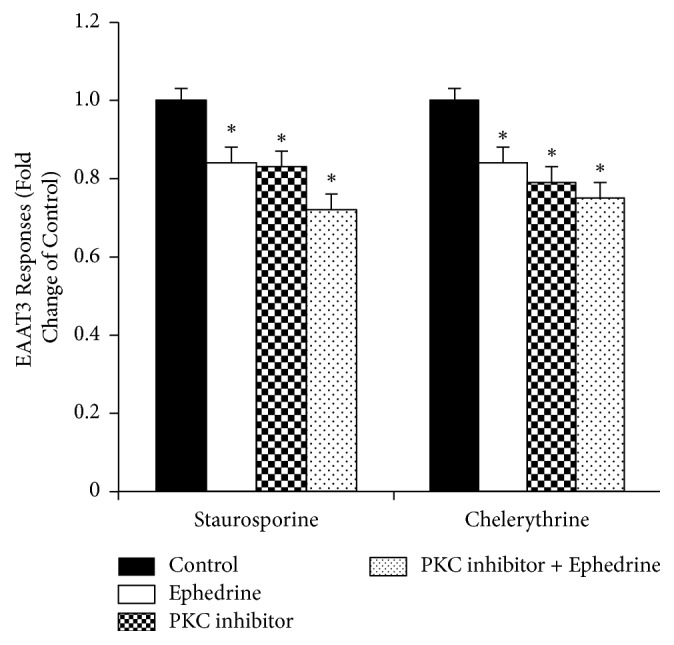
Effects of protein kinase C (PKC) inhibition on excitatory amino acid transporter type 3 (EAAT3) activity in the presence or absence of 1.19 *μ*M ephedrine. Whereas oocytes exposed to PKC inhibitor, ephedrine, or both showed a significant decrease in EAAT3 activity by 30 *μ*M glutamate compared with the control, the EAAT3 activities were not significantly different among oocytes treated with PKC inhibitor, ephedrine, or PKC inhibitor plus ephedrine. Data are means ± SEM,* n* = 31–46 for each data point. ^*∗*^*P* < 0.05 compared with the control.

Pretreatment of oocytes with the PKC inhibitors, staurosporine (2 *μ*M for 1 h) or chelerythrine (100 *μ*M for 1 h), reduced EAAT3 activity significantly (1.00 ± 0.03 for control versus 0.83 ± 0.04 for staurosporine,* n* = 37–41; or 1.00 ± 0.06 for control versus 0.79 ± 0.04 for chelerythrine,* n* = 31,* P* < 0.05). To verify whether there are interactions between the effects of these PKC inhibitors and aminophylline on EAAT3, staurosporine- or chelerythrine-treated oocytes were exposed to aminophylline, and the responses were compared. Oocytes exposed to aminophylline, PKC inhibitor (staurosporine or chelerythrine), or both showed significant decreases in EAAT3 activity compared with untreated controls. However, there were no significant differences in EAAT3 activities among oocytes treated with aminophylline, PKC inhibitor (staurosporine or chelerythrine), or PKC inhibitor plus aminophylline (Figure 5). Similarly, oocytes exposed to ephedrine, PKC inhibitor (staurosporine or chelerythrine), or both showed significant decreases in EAAT3 activity compared with untreated controls. Similarly, there were no significant differences among oocytes treated with ephedrine, PKC inhibitor (staurosporine or chelerythrine), or PKC inhibitor plus ephedrine (Figure 6).

**Figure 7:**
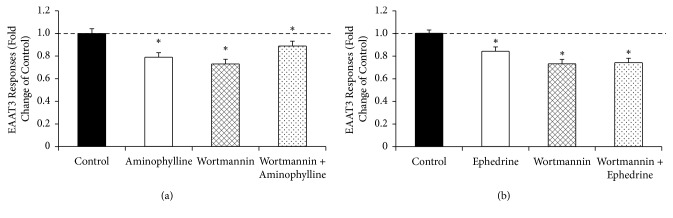
Effects of PI3K inhibition on excitatory amino acid transporter type 3 (EAAT3) activity in the presence or absence of 95.1 *μ*M aminophylline (a) and 1.19 *μ*M ephedrine (B). Whereas preincubation of oocytes with the PI3K inhibitor, wortmannin (1 *μ*M for 1 h), significantly reduced basal EAAT3 activity by 30 *μ*M glutamate, the activity did not differ among wortmannin, aminophylline, or both (a) and among wortmannin, ephedrine, or both (b). PI3K, phosphatidylinositol 3-kinase. Data are means ± SEM,* n* = 31–46 for each data point. ^*∗*^*P* < 0.05 compared with the control.

Oocytes pretreated with wortmannin, a PI3K inhibitor (10 *μ*M for 1 h), also showed significantly decreased EAAT3 activity (1.00 ± 0.03 for control versus 0.73 ± 0.04 for wortmannin,* n* = 34–39,* P* < 0.05). However, the activities were not significantly different among aminophylline, wortmannin, and wortmannin plus aminophylline groups, or among the ephedrine, wortmannin, and wortmannin plus ephedrine groups (Figures 7(a) and 7(b)).

**Figure 8:**
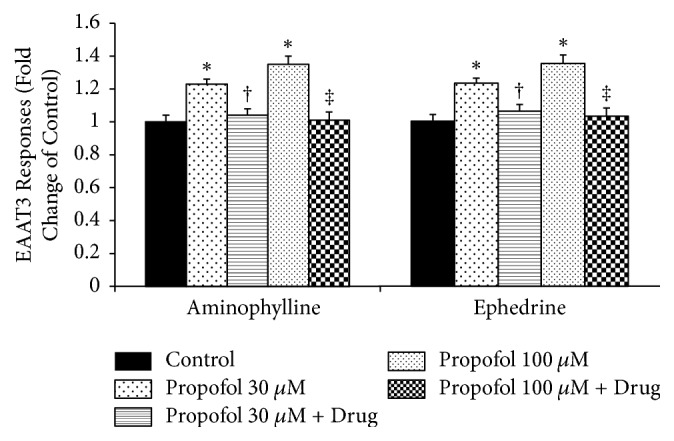
Reversible effects of aminophylline or ephedrine on enhanced activity of excitatory amino acid transporter type 3 (EAAT3) by propofol (30 and 100 *μ*M). Data are means ± SEM,* n* = 26–41 for each data point. ^*∗*^*P* < 0.05 compared with control; ^†^*P* < 0.05 compared with 30 *μ*M propofol; ^‡^*P* < 0.05 compared with 100 *μ*M propofol.

Propofol at 30 and 100 *μ*M significantly enhanced EAAT3 activity (1.00 ± 0.04 for control versus 1.23 ± 0.03 for propofol 30 *μ*M and 1.35 ± 0.05 for propofol 100 *μ*M,* P* < 0.05). However, there was no significant difference in EAAT3 activity between 30 *μ*M and 100 *μ*M propofol. These enhanced EAAT3 activities induced by 30 and 100 *μ*M propofol were significantly abolished by 95.1 *μ*M aminophylline (1.04 ± 0.04 for 30 *μ*M propofol + aminophylline; 1.01 ± 0.05 for 100 *μ*M propofol + aminophylline,* P* < 0.05 compared with each concentration of propofol) or 1.19 *μ*M ephedrine (1.06 ± 0.04 for 30 *μ*M propofol + ephedrine; 1.03 ± 0.05 for 100 *μ*M propofol + ephedrine,* P* < 0.05 compared with each concentration of propofol) (Figure 8).

## 4. Discussion

### 4.1. Effects of Flumazenil, Aminophylline, and Ephedrine on EAAT3 Activity

Our results showed that aminophylline (9.5–238 *μ*M) and ephedrine (595 nM and 1.19 *μ*M), but not flumazenil (0.016–1.319 M), decreased the activity of EAAT3.

The regulation of glutamate neurotransmission is a mechanism for anesthesia. A growing body of evidence suggests that changes in EAAT3 activity may be a novel mechanism of action of anesthetics [5, 6, 30]. Flumazenil, aminophylline, and ephedrine antagonize the sedative and anesthetic effects of anesthetics [8, 12–18]. Therefore, we expected that these drugs would alter the EAAT3 activity.

Aminophylline (theophylline ethylenediamine) is a nonselective adenosine receptor antagonist and contains 86% anhydrous theophylline [23]. The pharmacologically active, free extracellular brain concentration of theophylline is 75% of the blood concentration [31]. Considering that the therapeutic concentration of theophylline is 6–20 *μ*g/mL (28.5–95.1 *μ*M) [23], our results suggest that aminophylline can inhibit EAAT3 activity not only at clinically relevant concentrations but also at concentrations both lower and higher than the therapeutic ranges. This finding suggests a novel mechanism underlying the central effects of aminophylline, such as the excitatory effects on neuronal activity and CNS stimulation [10], proconvulsant effect [32, 33], and antagonism of the sedative effects of several anesthetics [12–15]. The central adenosine receptor antagonism, *γ*-aminobutyric acid A receptor blockade, and N-methyl-D-aspartate receptor activation may mediate the CNS stimulating effect of theophylline [34]. There is evidence of the involvement of adenosine neuromodulation in pentobarbital- or midazolam-induced depression of field excitatory postsynaptic potentials in rat hippocampal slices, which were partially or completely antagonized by aminophylline, respectively [35, 36]. These findings support our results.

Ephedrine, an indirect sympathomimetic drug, is widely used to treat hypotension and is a potent stimulator of the CNS leading to insomnia as a common CNS side effect [37]. Ephedrine has neurotoxic potential, including increased microdialysate levels of dopamine, serotonin, and glutamate [38], and increases the MAC of halothane and BIS during sevoflurane or propofol anesthesia [16–18]. Therefore, we postulated that ephedrine may alter the EAAT3 activity as a mechanism of CNS stimulating action.

The single dose peak level of ephedrine is 40–140 ng/mL [24], and therapeutic range used for bronchodilation is 20–80 ng/mL [25]. However, in a clinical study using continuous infusion of ephedrine to treat hypotension, the interquartile range of arterial concentrations of ephedrine was 306.5–523.5 ng/mL after injection of ephedrine at a dose of 44.8–79.2 mg [26]. Therefore, we tested a wide range of ephedrine concentrations from 50 nM (10 ng/mL) to 4.96 *μ*M (1000 ng/mL). In the present study, ephedrine decreased EAAT3 activity at two concentrations, that is, 595 nM (120 ng/mL) and 1.19 *μ*M (240 ng/mL). These are within the concentration range seen under clinical conditions, considering that ephedrine is used to treat hypotension at repeat doses of 0.1 mg/kg or continuous infusion [17, 18, 26]. The reason for the biphasic ephedrine dose-response pattern on EAAT3 activity is not clear. Although the release of norepinephrine and dopamine may be responsible for increasing the BIS after ephedrine injection [17], the effect of ephedrine on EAAT3 activity may also play a role in antagonizing the anesthetic action.

Flumazenil had no effect on EAAT3 activity at any concentration used. Although several reports have suggested that flumazenil has intrinsic actions, such as reversing hepatic coma and increasing anxiety [7, 8], their mechanisms are unlikely to be mediated by decreased EAAT3 activity. In one study, flumazenil reversed the anticonvulsant properties of diazepam against lidocaine-induced convulsions, but itself had no effect on lidocaine-induced convulsions in rats [39]. In another study, lidocaine at 100 *μ*M and 1 mM enhanced EAAT3 activity, suggesting a mechanism of proconvulsant action of lidocaine [40]. Taken together, flumazenil itself may not affect EAAT3 activity, consistent with our results.

The results of kinetic analyses showed that both 95.1 *μ*M aminophylline and 1.19 *μ*M ephedrine decreased *V*_max_, but not *K*_*m*_, of EAAT3 for glutamate, suggesting that aminophylline and ephedrine decrease the availability or turnover rate of EAAT3 rather than lowering the affinity of EAAT3 to glutamate.

### 4.2. Involvement of PKC and PI3K in the Effects of Aminophylline and Ephedrine on EAAT3 Activity

In the mechanism study, we demonstrated that PKC activation by PMA enhanced the activity of EAAT3, consistent with previous reports [5, 6], and this PMA-induced increase in EAAT3 activity was abolished by aminophylline or ephedrine. We also showed that two PKC inhibitors (staurosporine and chelerythrine) and a PI3K inhibitor (wortmannin) significantly reduced the EAAT3 activity, consistent with previous reports [41, 42] and there were no significant differences in EAAT3 activities among PKC inhibitors or PI3K inhibitor, aminophylline or ephedrine, and PKC inhibitors or PI3K inhibitor + aminophylline or ephedrine groups. That is, there were no additive or synergistic interactions between PKC inhibitors or PI3K inhibitor and aminophylline or ephedrine. Therefore, our results suggest that the decreased activity of EAAT3 by aminophylline or ephedrine is mediated by PKC and PI3K.

The activation of PKC and PI3K increased cell-surface expression of EAAT3 [43]. The increased EAAT3 activity by PMA-induced PKC activation in Xenopus oocytes may be mediated via the increase in EAAT3 cell-surface expression [44, 45]. Our kinetic data suggested that aminophylline and ephedrine affected the cell-membrane expression of EAAT3.

### 4.3. Interactions between the Effects of Aminophylline or Ephedrine and Propofol on EAAT3 Activity

In this study, propofol at concentrations of 30 and 100 *μ*M significantly increased EAAT3 activity; these enhanced activities of EAAT3 were significantly abolished by aminophylline or ephedrine.

The increased EAAT3 activity caused by propofol may be a mechanism for propofol-induced anesthesia, anticonvulsive effects, and neuroprotection [6]. The blood concentrations of propofol at which 95% of patients did not respond to verbal commands and to skin incision were 5.4 and 27.4 *μ*g/mL, respectively, when used alone [46]. The optimal effect-site (brain) concentrations of propofol with 95% probability of no response to surgical stimuli were 2.5–5.5 *μ*g/mL (14–31 *μ*M) when administered with opioids [47].

The increased EAAT3 activities by 30 and 100 *μ*M propofol were abolished by 95.1 *μ*M aminophylline or 1.19 *μ*M ephedrine. The findings that propofol-induced increased EAAT3 activities were abolished by aminophylline and ephedrine also suggest that aminophylline and ephedrine reverse the upregulation of EAAT3 activity by propofol, which may be a mechanism of aminophylline reversal of prolonged sedation after propofol anesthesia [15], of pharmacodynamic antagonistic interaction of aminophylline with propofol [48], and of the increase in BIS by ephedrine during propofol anesthesia [18].

To determine the interactions between the effects of aminophylline or ephedrine and propofol on EAAT3 activity in our study, these two concentrations of propofol were selected based on the result of a previous report [6], in which 30 and 100 *μ*M of propofol enhanced the EAAT3 activity. Considering high plasma protein binding of propofol (97–99%), even 30 *μ*M of propofol concentration used in this study may be considered too high to extend our results to clinical situation because unbound free propofol in brain exerts the effect. Although measurement of propofol concentration in human brain tissue remains impossible, Dawidowicz et al. [49] and Engdahl et al. [50] measured the propofol concentration in human cerebrospinal fluid (CSF) in neurosurgical patients as a surrogate for the propofol concentration in brain tissue. Engdahl et al. reported that the propofol concentration in CSF was 1.6% of the plasma concentration. Dawidowicz et al. showed that the unbound propofol concentration in plasma is 1.12% of the total concentration in plasma and free propofol concentration in CSF is 61.8% of the free propofol concentration in plasma. However, it is not considered that their results correspond to the propofol concentration in brain tissues exactly, in consideration of some animal research findings that the brain/plasma ratio of propofol in the rat is 7.8–8.5 [51] and the brain/artificial CSF partition coefficient in rat brain is 36 [52].

This study has several limitations. First, it was an* in vitro* study, and rat EAAT3 mRNA was used instead of human mRNA. Therefore, it may have poor translatability to cells in the CNS or in humans. Thus, further studies are required to replicate or verify our results. Second, EAAT3 may not be a major target for anesthetics. However, there is a growing body of evidence suggestive of a role for EAAT3 in the mechanism for various effects of anesthetics. Additionally, this is the first study to investigate the drug interactions (between propofol and aminophylline or ephedrine) with regard to EAAT3 activity, and our results reflect the opposite effects. Third, we chose 95.1 *μ*M aminophylline for further experiments after the dose-response study. This concentration may be slightly higher than the upper limit of the therapeutic range of theophylline considering that aminophylline contains 86% anhydrous theophylline (81.8 *μ*M) and that the free extracellular brain concentration of theophylline is 75% of the blood concentration {71.3 *μ*M = 0.75 × 95.1 *μ*M (upper limit of therapeutic range of theophylline)}. However, the results may be similar at lower concentrations because the degrees of inhibition on EAAT3 activity were similar (16–24%) at all concentrations of aminophylline studied.

## 5. Conclusions

In conclusion, aminophylline and ephedrine, but not flumazenil, inhibited EAAT3 activity at clinically relevant concentrations. PKC and PI3K seem to be involved in these effects. Aminophylline and ephedrine abolished the increase in EAAT3 activity by propofol. Our results indicate a novel site of action for aminophylline and ephedrine to increase glutamatergic neurotransmission.

## References

[1] Danbolt N. C. (2001). Glutamate uptake. *Progress in Neurobiology*.

[2] Kanai Y., Hediger M. A. (1992). Primary structure and functional characterization of a high-affinity glutamate transporter. *Nature*.

[3] Kanai Y., Smith C. P., Hediger M. A. (1993). A new family of neurotransmitter transporters: The high-affinity glutamate transporters. *The FASEB Journal*.

[4] Sepkuty J. P., Cohen A. S., Eccles C. (2002). A neuronal glutamate transporter contributes to neurotransmitter GABA synthesis and epilepsy. *The Journal of Neuroscience*.

[5] Do S.-H., Kamatchi G. L., Washington J. M., Zuo Z. (2002). Effects of volatile anesthetics on glutamate transporter, excitatory amino acid transporter type 3: The role of protein kinase C. *Anesthesiology*.

[6] Do S.-H., Ham B.-M., Zuo Z. (2003). Effects of propofol on the activity of rat glutamate transporter type 3 expressed in Xenopus oocytes: the role of protein kinase C. *Neuroscience Letters*.

[7] Bansky G., Meier P. J., Ziegler W. H., Walser H., Schmid M., Huber M. (1985). Reversal of hepatic coma by benzodiazepine antagonist (Ro 15-1788). *The Lancet*.

[8] Schöpf J., Laurian S., Le P. K., Gaillard J.-M. (1984). Intrinsic activity of the benzodiazepine antagonist Ro 15-1788 in man: An electrophysiological investigation. *Pharmacopsychiatry*.

[9] Dunwiddie T. V. (1985). The physiological role of adenosine in the central nervous system. *International Review of Neurobiology*.

[10] Porkka-Heiskanen T. (1999). Adenosine in sleep and wakefulness. *Annals of Medicine*.

[11] Tung A., Herrera S., Szafran M. J., Kasza K., Mendelson W. B. (2005). Effect of sleep deprivation on righting reflex in the rat is partially reversed by administration of adenosine A1 and A2 receptor antagonists. *Anesthesiology*.

[12] Arvidsson S., Niemand D., Martinell S., Ekström‐Jodal B. (1984). Forum Aminophylline reversal of diazepam sedation. *Anaesthesia*.

[13] Krintel J. J., Wegmann F. (1987). Aminophylline reduces the depth and duration of sedation with barbiturates. *Acta Anaesthesiologica Scandinavica*.

[14] Turan A., Memis D., Karamanlioğlu B., Çolak A., Pamukçu Z., Turan N. (2002). Effect of aminophylline on recovery from sevoflurane anaesthesia. *European Journal of Anaesthesiology*.

[15] Sakurai S., Fukunaga A., Fukuda K., Kasahara M., Ichinohe T., Kaneko Y. (2008). Aminophylline reversal of prolonged postoperative sedation induced by propofol. *Journal of Anesthesia*.

[16] Steffey E. P., Eger E. I. (1975). The effect of seven vasopressors on halothane MAC in dogs. *British Journal of Anaesthesia*.

[17] Ishiyama T., Oguchi T., Iijima T., Matsukawa T., Kashimoto S., Kumazawa T. (2003). Ephedrine, but not phenylephrine, increases bispectral index values during combined general and epidural anesthesia. *Anesthesia & Analgesia*.

[18] Takizawa D., Takizawa E., Miyoshi S. (2006). The effect of ephedrine and phenylephrine on BIS values during propofol anaesthesia. *European Journal of Anaesthesiology*.

[19] Borycz J., Pereira M. F., Melani A. (2007). Differential glutamate-dependent and glutamate-independent adenosine A 1 receptor-mediated modulation of dopamine release in different striatal compartments. *Journal of Neurochemistry*.

[20] Woo J. H., Han J. I., Baik H. J., Lee H. (2012). Effects of clonidine on the activity of the rat glutamate transporter EAAT3 expressed in Xenopus oocytes. *Korean Journal of Anesthesiology*.

[21] Kim J.-H., Lim Y.-J., Ro Y.-J. (2003). Effects of ethanol on the rat glutamate excitatory amino acid transporter type 3 expressed in Xenopus oocytes: Role of protein kinase C and phosphatifylinositol 3-kinase. *Alcoholism: Clinical and Experimental Research*.

[22] Coleman R. L., Temo J., White P. F. (1997). Benzodiazepines. *Textbook of Intravenous Anesthesia*.

[23] Rall T. W., Gilman A. G., Rall T. W., Nies A. S., Taylor P. (1991). Drugs used in the treatment of asthma. The methylxanthines, cromolyn sodium, and other agents. *Goodman & Gilman’s the pharmacological basis of therapeutics*.

[24] Ohtsuji M., Lai J. S., Binder S. R., Kondo T., Takayasu T., Ohshima T. (1996). Use of REMEDi HS in emergency toxicology for a rapid estimate of drug concentrations in urine, serum, and gastric samples. *Journal of Forensic Sciences*.

[25] Haller C. A., Benowitz N. L. (2000). Adverse cardiovascular and central nervous system events associated with dietary supplements containing ephedra alkaloids. *The New England Journal of Medicine*.

[26] Ngan Kee W. D., Khaw K. S., Tan P. E., Ng F. F., Karmakar M. K. (2009). Placental transfer and fetal metabolic effects of phenylephrine and ephedrine during spinal anesthesia for cesarean delivery. *Anesthesiology*.

[27] Baik H.-J., Lee S.-A., Washington J. M., Zuo Z.-Y. (2009). Amitriptyline inhibits the activity of the rat glutamate transporter EAAT3 expressed in Xenopus oocytes. *Journal of Pharmacy and Pharmacology*.

[28] Kim J.-H., Do S.-H., Kim Y.-L., Zuo Z. (2005). Effects of chronic exposure to ethanol on glutamate transporter EAAT3 expressed in Xenopus oocytes: Evidence for protein kinase C involvement. *Alcoholism: Clinical and Experimental Research*.

[29] Na H.-S., Park H.-P., Kim C.-S., Do S.-H., Zuo Z., Kim C.-S. (2012). 17*β*-Estradiol attenuates the activity of the glutamate transporter type 3 expressed in Xenopus oocytes. *European Journal of Pharmacology*.

[30] Palmada M., Böhmer C., Centelles J. J., Kinne R. K. H. (1999). Effect of benzodiazepines on the epithelial and neuronal high-affinity glutamate transporter EAAC1. *Journal of Neurochemistry*.

[31] Ståhle L., Segersvärd S., Ungerstedt U. (1991). Drug distribution studies with microdialysis II. Caffeine and theophylline in blood, brain and other tissues in rats. *Life Sciences*.

[32] Bernaskova K., Mares P. (2000). Proconvulsant effect of aminophylline on cortical epileptic afterdischarges varies during ontogeny. *Epilepsy Research*.

[33] Stern L., Dannon P. N., Hirschmann S. (1999). Aminophylline increases seizure length during electroconvulsive therapy. *The Journal of ECT*.

[34] Amabeoku G. J. (1999). Gamma-aminobutyric acid and glutamic acid receptors may mediate theophylline-induced seizures in mice. *General Pharmacology: The Vascular System*.

[35] Tohdoh Y., Narimatsu E., Kawamata M., Namiki A. (2000). The involvement of adenosine neuromodulation in pentobarbital-induced field excitatory postsynaptic potentials depression in rat hippocampal slices. *Anesthesia & Analgesia*.

[36] Narimatsu E., Niiya T., Kawamata M., Namiki A. (2006). The mechanisms of depression by benzodiazepines, barbiturates and propofol of excitatory synaptic transmissions mediated by adenosine neuromodulation. *The Japanese Journal of Anesthesiology*.

[37] Westfall T. C., Westfall D. P., Brunton L. L., Lazo J. S., Parker K. L. (2006). Adrenergic agonists and antagonists. *Goodman & Gilman’s the pharmacological basis of therapeutics*.

[38] Bowyer J. F., Newport G. D., Slikker W., Gough B., Ferguson S. A., Tor-Agbidye J. (2000). An evaluation of l-ephedrine neurotoxicity with respect to hyperthermia and caudate/putamen microdialysate levels of ephedrine, dopamine, serotonin, and glutamate. *Toxicological Sciences*.

[39] Yokoyama M., Benson K. T., Arakawa K., Goto H. (1992). Effects of flumazenil on intravenous lidocaine-induced convulsions and anticonvulsant property of diazepam in rats. *Anesthesia & Analgesia*.

[40] Do S.-H., Fang H.-Y., Ham B.-M., Zuo Z. (2002). The effects of lidocaine on the activity of glutamate transporter EAAT3: The role of protein kinase C and phosphatidylinositol 3-kinase. *Anesthesia & Analgesia*.

[41] Gil Y. S., Kim J. H., Kim C. H., Han J. I., Zuo Z., Baik H. J. (2015). Gabapentin inhibits the activity of the rat excitatory glutamate transporter 3 expressed in Xenopus oocytes. *European Journal of Pharmacology*.

[42] Shin H.-J., Ryu J.-H., Kim S.-T., Zuo Z., Do S.-H. (2013). Caffeine-induced inhibition of the activity of glutamate transporter type 3 expressed in Xenopus oocytes. *Toxicology Letters*.

[43] Beart P. M., O'Shea R. D. (2007). Transporters for L-glutamate: An update on their molecular pharmacology and pathological involvement. *British Journal of Pharmacology*.

[44] González M. I., Kazanietz M. G., Robinson M. B. (2002). Regulation of the neuronal glutamate transporter excitatory amino acid carrier-1 (EAAC1) by different protein kinase C subtypes. *Molecular Pharmacology*.

[45] Johnson J., Capco D. G. (1997). Progesterone acts through protein kinase C to remodel the cytoplasm as the amphibian oocyte becomes the fertilization-competent egg. *Mechanisms of Development*.

[46] Smith C., McEwan A. I., Jhaveri R. (1994). The interaction of fentanyl on the Cp50 of propofol for loss of consciousness and skin incision. *Anesthesiology*.

[47] Vuyk J., Mertens M. J., Olofsen E., Burm A. G. L., Bovill J. G. (1997). Propofol anesthesia and rational opioid selection: Determination of optimal EC50-EC95 propofol- opioid concentrations that assure adequate anesthesia and a rapid return of consciousness. *Anesthesiology*.

[48] Lee S.-H., Kang H.-J., Jin S.-J. (2014). Impact of aminophylline on the pharmacodynamics of propofol in beagle dogs. *Journal of Pharmacokinetics and Pharmacodynamics*.

[49] Dawidowicz A. L., Kalitynski R., Fijalkowska A. (2004). Relationships between total and unbound propofol in plasma and CSF during continuous drug infusion. *Clinical Neuropharmacology*.

[50] Engdahl O., Abrahams M., Björnsson A. (1998). Cerebrospinal fluid concentrations of propofol during anaesthesia in humans. *British Journal of Anaesthesia*.

[51] Shyr M.-H., Tsai T.-H., Tan P. P. C., Chen C.-F., Chan S. H. H. (1995). Concentration and regional distribution of propofol in brain and spinal cord during propofol anesthesia in the rat. *Neuroscience Letters*.

[52] Gredell J. A., Turnquist P. A., MacIver M. B., Pearce R. A. (2004). Determination of diffusion and partition coefficients of propofol in rat brain tissue: Implications for studies of drug action in vitro. *British Journal of Anaesthesia*.

